# Miniemulsion polymerization as a versatile tool for the synthesis of functionalized polymers

**DOI:** 10.3762/bjoc.6.130

**Published:** 2010-12-01

**Authors:** Daniel Crespy, Katharina Landfester

**Affiliations:** 1 Max Planck Institute for Polymer Research, Ackermannweg 10, 55128 Mainz, Germany

**Keywords:** functionalized polymers, heterophase polymerization, miniemulsion, polymer colloids, polymerization

## Abstract

The miniemulsion technique is a particular case in the family of heterophase polymerizations, which allows the formation of functionalized polymers by polymerization or modification of polymers in stable nanodroplets. We present here an overview of the different polymer syntheses within the miniemulsion droplets as reported in the literature, and of the current trends in the field.

## Review

### Introduction

Miniemulsions are a special class of emulsions that are stabilized against coalescence (by a surfactant) and Ostwald ripening (by an osmotic pressure agent). The miniemulsions are produced by high-energy homogenization and usually yield stable and narrowly distributed droplets with a size ranging from 50 to 500 nm. Apolar droplets can be dispersed in a polar liquid to give direct miniemulsions (most classically oil-in-water), whereas the contrary (polar droplets in a non-polar liquid) leads to inverse miniemulsions (e.g., water-in-oil, w/o). Water-free miniemulsions can be formed in direct or inverse systems. Polymers can be synthesized in a miniemulsion system in the dispersed phase, at the interface of the droplets, or in the continuous phase, although only the two first possibilities are usually found in the literature. In principle, the synthesis of functionalized polymers in miniemulsion can occur either by (co)polymerizing one or several functional monomers, or by the modification of polymers present in the dispersed phase of a miniemulsion. In the latter case, the functionalization can occur by the reaction or the assembly of small molecules on the polymer, by the grafting of macromolecules or by degradation of the polymer. The functionalized polymer originates therefore from one or several reactive monomers or is the product of the transformation of a non-functional polymer to a functional polymer. The IUPAC recommendation for the term “functional polymer” is very broad since it includes polymers bearing “specified chemical groups” and polymers having “specified physical, chemical, biological, pharmacological, or other uses which depend on specific chemical groups” [1]. Reviews on miniemulsion have recently been published, focusing on the kinetics of polymerization [2–3], the structure of the obtained nanoparticles [4], and their applications in medicine [5] as well as for catalysis [6]. As a consequence of the mechanism of the formation of miniemulsions, and due to their colloidal properties and stability, a large range of different polymers colloids can be generated using a miniemulsion. Table 1 shows some important references for the synthesis of commercially important polymers.

**Table 1:** Important polymer classes produced by miniemulsion polymerization.

Polymer class	Polymerization	Year	Ref.

polystyrene	radical	1973	[7]
polyvinyl chloride	radical	1984	[8]
silicone	anionic	1994	[9]
polyethylene	catalytic	2000	[10]
epoxy	polyaddition	2000	[11]
polyurethane	polyaddition	2001	[12]
saturated polyester	polycondensation	2003	[13]
polyamide	anionic	2005	[14]
polyimide	polycondensation	2009	[15]

In this review, we will focus on the description of the possibilities offered by the miniemulsion process to carry out chain polymerization, polyaddition, polycondensation, and modifications of polymers, and the trends followed in this research field. The so-called “artificial miniemulsions”, i.e., the miniemulsion of preformed polymer, are not described in this review.

### Free-radical polymerization

Most of the reported polymer syntheses in miniemulsion are performed via free-radical polymerization. In fact, the polymerization is very simple to perform and yields are usually high. Moreover, the polydispersity in size of the miniemulsion particles and the dispersity of the polymer are not directly correlated, and for applications the focus is in many cases on the nanoparticles themselves, i.e., size and size distribution or morphology, and not on the characteristics of the polymer chains (for a given polymer). Functionalized polymers can be obtained by the homopolymerization of a functional monomer or its copolymerization with another (non-functional) monomer. The generation of functional (and therefore often hydrophilic) (homo)polymer particles in inverse miniemulsion is more straightforward than direct miniemulsions, since the presence of functional groups such as amino, hydroxy, or carboxylic acid groups tends to increase the hydrophilicity of the monomer. The polymerization of hydrophilic monomers in inverse miniemulsions was recently reviewed by Capek [16]. The functional groups that can be introduced in latexes by free-radical polymerization in inverse miniemulsion are overviewed in Table 2.

**Table 2:** Functional monomers or comonomers polymerized in inverse miniemulsion (crosslinkers are not mentioned here).

Functional group	(co)Monomer	Ref.

carboxylic acid	methacrylic acid	[17,24]
	acrylic acid	[18–20,23]
		
hydroxy	2-hydroxyethyl methacrylate	[18,21]
	vinyl gluconamide	[28]
		
sulfonate	2-acrylamido-2-methyl- 1-propanesulfonic acid	[25–26]

Homopolymers of crosslinked polymethacrylic acid [17], polyacrylic acid, i.e., poly(acrylic acid) and poly(acrylic acid sodium salt) [18–20], and poly(2-hydroxyethyl methacrylate) [18,21] were synthesized in inverse w/o miniemulsions. The polymerization of 2-hydroxyethyl methacrylate could be performed in inverse miniemulsion with a surface active initiator or an oil-soluble initiator. The initiator potassium peroxodisulfate (KPS) could not be used due to its low solubility in the 2-hydroxyethyl methacrylate monomer. Similarly, sodium acrylate was polymerized in inverse miniemulsion and was crosslinked with diethylene glycol diacrylate to yield, after the transfer to water, stable microgels. Monodisperse latexes could be obtained with the poly(2-hydroxyethyl methacrylate) when water, methanol, ethanol, ethylene glycol, or water/ethanol mixtures were used as the dispersed phase in the presence of cobalt tetrafluoroborate [21]. This approach is particularly interesting to encapsulate large amounts of metal salts. Indeed, up to 22.6 wt % of the cobalt salt compared to the monomer content could be encapsulated. Monodisperse latexes of silver–polymer particles were also obtained upon reduction of silver nitrate in the monomer droplets followed by polymerization of the dispersed phase [22]. The reduction reaction was performed at high temperature and miniemulsions were kept stable. This novel system opens up the area of high temperature reactions in miniemulsion. Acrylic acid was also copolymerized with trivinylacrylic acid in inverse minemulsion [23]. The non-reacted vinyl groups, detected by NMR spectroscopy, could be subsequently further crosslinked. The syntheses of temperature-responsive microgels in inverse miniemulsion have also been reported [24]. *N*-isopropylacrylamide and methacrylic acid were polymerized in the presence of Fe_3_O_4_ ferrofluid to yield superparamagnetic particles. Wiechers et al. investigated the copolymerization of 1-vinylimidazole and 2-acrylamido-2-methyl-1-propanesulfonic acid in inverse miniemulsion with an oil-soluble initiator at different pH values [25]. The polymerization was found to be faster at neutral pH values and higher molecular weight polymers were produced in miniemulsion compared to solution polymerization as a result of the confinement effect. The same group carried out a similar study with the copolymerization of 2-acrylamido-2-methyl-1-propanesulfonic acid and 2-(dimethylamino)ethyl methacrylate [26].

**Figure 1:**
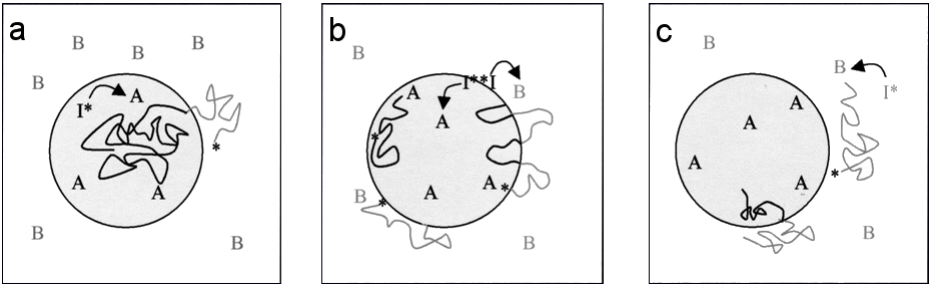
Copolymerization of 2 monomers A and B with different polarities in direct miniemlusions with the decomposed initiators I^*^ which are **a:** oil-soluble, **b:** surface active, **c:** water-soluble (reproduced with permission from [27]. Copyright (2002) Wiley-VCH Verlag GmbH & Co, KGaA).

**Figure 2:**
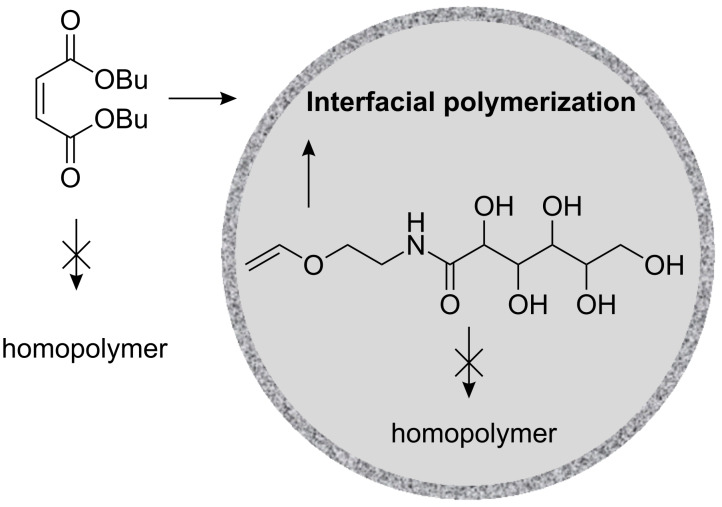
Interfacial alternating radical copolymerization between dibutyl maleate and vinyl gluconamide for building capsules (from [28]).

In the copolymerization approaches discussed previously, the comonomers were present in the dispersed phase. Willert et al. studied the copolymerization of monomers with opposite polarity, i.e., one comonomer in each phase, in direct and inverse miniemulsion [27]. Water-soluble, surface active, and oil-soluble initiators were employed to initiate the polymerizations as shown in Figure 1. Oil-soluble initiators were found to give a higher yield of copolymers of acrylamide and methyl methacrylate with a low extent of blockiness than with a water-soluble initiator or surface active initiator. By contrast, the surface active polyethylene glycol azo-initiator yielded polymers almost free of homopolymers with a low blockiness when acrylamide and styrene were copolymerized. Wu et al. used the same principle but with monomers having the ability to copolymerize alternately [28]. Thus water-soluble poly(hydroxy vinyl ether)s were copolymerized with oil-soluble maleate esters to yield polymer particles with capsular morphology as shown in Figure 2.

While the formation of (hydrophilic) functionalized particles is straightforward in inverse miniemulsions, it most often required the presence of a second hydrophobic monomer when the polymerization was performed in direct miniemulsions. The hydrophilic acrylic acid or methacrylic acid was successfully polymerized in direct miniemulsion with the hydrophobic octadecyl methacrylate to yield functionalized comb-like polymers [29]. Acrylic acid was copolymerized with styrene to improve the hydrophilicity of the resultant polymer with phase separation of the polymer in oil nanodroplets, to form nanocapsules (and not hemispheres or separate nanoparticles of each phase) [30]. Particles with capsular morphologies suitable for the encapsulation of hydrophobic substances could be obtained. Capsules could be also obtained when methacrylic acid was used instead of acrylic acid [31]. Wu and Schork investigated the copolymerization between the functional *n*-methylol acrylamide and vinyl acetate in batch and semi-batch processes [32]. For a batch process with an initiator in the aqueous phase, it was found that the copolymerization followed the Mayo–Lewis equation despite the huge difference of solubility of the monomers in the aqueous continuous phase. Fluorinated monomers (fluoroalkyl acrylates) could be easily polymerized when dispersed in water in a miniemulsion system [33]. These monomers are typically difficult to polymerize in traditional emulsion polymerizations because of the very low water solubility of the monomers and the oligomers. Functionality was introduced via copolymerization with protonated monomers such as acrylic acid and methacryloxyethyltrimethyl ammonium chloride (MADQUAT).

Styrene was copolymerized with various functional comonomers to be used as model systems for nanoparticles–cell interaction. In fact, a fluorescent marker suitable for FACS and LSM measurements could be encapsulated in the monodisperse nanoparticles with a controlled density of functionalization of the surface. Thus, the influence of the surface functionality nature and density of the nanoparticles on their uptake by different cell lines could be investigated. 2-Aminoethyl methacrylate (0–20 wt %) or acrylic acid were copolymerized with styrene to yield functionalized particles [34–35] and their uptake by cells was studied [34]. In general, with increased functional groups, an increase in the uptake into cells could be observed. Copolymer particles of styrene and acrylic acid were used to encapsulate a platinum(II) complex for photolithographic applications [36], magnetite [37], and as templates for the mineralization on the surface of particles [38]. Ethirajan et al. showed, for instance, that it was possible to use the surface of nanoparticles with carboxylate groups and calcium counterions to mineralize hydroxyapatite on the surface of the particles. Methyl methacrylate, butyl acrylate, and acrylic acid were copolymerized in the presence of alkyd resins to yield hybrid latexes [39]. Similar copolymerizations were carried out with styrene, tetraethylene glycol diacrylate as crosslinker, and up to 20 mol % of 2-hydroxyethyl methacrylate, 2-aminoethyl methacrylate or styrene sulfonic acid [40]. 2-Hydroxyethyl methacrylate and 2-hydroxypropyl methacrylate were copolymerized with styrene and the influence of the functional comonomers on the nucleation mechanism was investigated [41]. A mixture of styrene, butyl acrylate, and butyl methacrylate was also copolymerized with 2-aminoethyl methacrylate by the same authors [42]. Cell–particle investigations were performed with polyisoprene and copolymers of styrene and isoprene fluorescent particles. Their uptake was found to be faster in comparison to polystyrene particles [43]. Such particles can be considered as reactive latexes since they can be easily crosslinked in a subsequent step due to the presence of the double bond. In the case of vinylphosphonic acid, the monomer was added to styrene or MMA with an oil-soluble initiator to form the dispersed phase [44]. In contrast to latexes with styrene as monomer, the use of MMA led to an increase of coagulum by increasing the amount of vinylphosphonic acid. This is explained by the higher solubility of MMA in water as compared to styrene which leads to polymerization of MMA in the continuous phase via homogeneous nucleation. On the other hand, latexes with MMA and 10 wt % vinylphosphonic acid showed the highest density of phosphonate functionality (0.66 group/nm²) at pH = 10. Lu et al. introduced the term “emulsifier-free miniemulsion polymerization” for the copolymerization of styrene and sodium *p*-styrene sulfonate in the presence of magnetite stabilized by oleic acid [45]. The term “emulsifier-free” is debatable since oleic acid possesses surface activity. The concept of copolymerizing with a functional comonomer that is soluble in the continuous phase can be obviously extended to virtually any vinyl functional monomers provided that the copolymerization parameters under such conditions allow copolymerization. The functionalities available using hydrophobic monomers with functional monomers in direct miniemulsions are summarized in Table 3.

**Table 3:** Functional comonomers employed in polymerizations with styrene, (meth)acrylates, and vinyl acetate in direct miniemulsion.

Main monomer	Functional comonomer	Functional group	Ref.

styrene	acrylic acid	carboxylic acid	[30,34–38]
	methacrylic acid	carboxylic acid	[31]
	2-aminoethyl methacrylate	amino	[34–35,40]
	isoprene	double bond	[43]
	vinylphosphonic acid	phosphonate	[44]
	2-hydroxyethyl methacrylate	hydroxy	[40–41]
	2-hydroxypropyl methacrylate	hydroxy	[41]
	styrene sulfonic acid	sulfonate	[40,45]
methyl methacrylate	vinylphosphonic acid	phosphonate	[44]
styrene, butyl acrylate, butyl methacrylate	2-aminoethyl methacrylate	amino	[42]
methyl methacrylate, butyl acrylate	acrylic acid	carboxylic acid	[39]
vinyl acetate	*n*-methylol acrylamide	hydroxy	[32]
octadecyl methacrylate	acrylic acid, methacrylic acid		[29]
fluoroalkyl acrylates	acrylic acid	carboxylic acid	[33]
	methacryloxyethyltrimethyl ammonium chloride	ammonium	[33]

We have already mentioned above that latexes can be prepared from polyisoprene and that these can be further crosslinked. Latexes with double functionality were also prepared by the free-radical polymerization of divinylbenzene in miniemulsion [46–47]. The remaining vinyl group after polymerization could be, for instance, reacted with thiol-functionalized PEG via the thiol–ene chemistry [47].

### Controlled radical polymerizations

Controlled radical polymerization techniques are suitable for synthesizing polymers with a high level of architectural control. They not only allow the copolymerization with functional monomers as discussed previously for free-radical polymerization, but also simple functionalization of the chain end by the initiator. Miniemulsion systems were found to be suitable to conduct controlled radical polymerizations [48–51] including atom transfer radical polymerization (ATRP), reversible addition fragmentation transfer (RAFT), degenerative iodine transfer [48], and nitroxide mediated polymerization (NMP). ATRP in miniemulsions was recently described in several reviews [52–53]. The kinetics of RAFT polymerization in miniemulsion has been discussed by Tobita [54] and thus no detailed description is required here.

### *Sur*factant mono*mer* (surfmer) in radical miniemulsion polymerization

**Figure 3:**
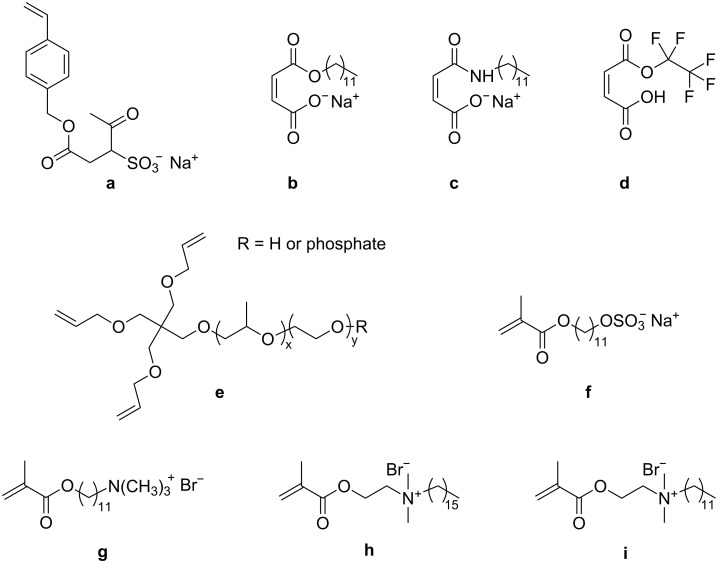
Chemical structures of the surfmers for radical polymerization in miniemulsions: **a:** sodium vinylbenzylsulfosuccinate [55], **b:** hemiester and **c:** hemiamide derivatives of maleic acid [56], **d:** mono-fluorooctyl maleate [57], **e:** PEO/PPO containing surfactant [46], **f:** 11-methacryloyloxyundecan-1-yl sulfate [58], **g:** 11-methacryloyloxyundecan-1-yl trimethyl ammonium bromide [58], **h:** *N,N*-dimethyl-*N*-*n*-hexadecyl-*N*-methacryloyloxyethyl ammonium bromide [59], **i:** *N*,*N*-dimethyl-*N*-*n*-dodecyl-*N*-methacryloyloxyethyl ammonium bromide [60].

The presence of the surfactant used to stabilize the latexes can have an unfavorable effect on surface properties of films prepared from these latexes. Therefore the use of copolymerizable surfactants was investigated for a number of different polymerization systems. For some step-growth polymerizations in inverse miniemulsions, the surfactant is incorporated into the particle as a result of the end functional groups. Because there is usually only one reactive group in the surfactant, reaction with the surfactant is detrimental for the molecular weight of the polymer. We will focus here only on radical polymerization processes, for which the copolymerization is directed by the copolymerization parameters of the monomers. The functionalities available from the literature are listed in Table 4 and the chemical structures are displayed in Figure 3.

**Table 4:** Functionalities of polymerizable surfactants used for the stabilization miniemulsions.

Functionality	Ref.

sulfosuccinate	[55]
sulfate	[58]
carboxylate	[56]
phosphate	[46]
hydroxy	[46]
fluorinated	[57]
quaternary ammonium	[58–60]

Styrene and methyl methacrylate were copolymerized with the sodium salt of vinylbenzylsulfosuccinic acid [55]. Stable latexes were obtained with oil-soluble or water-soluble initiators. The authors estimated that 50 to 75% of the surfactant was efficiently grafted onto the surface of the particles. A polymerizable surfactant was formed by the esterification of hydroxypropyl methacrylate or hydroxyethyl methacrylate with succinic anhydride [56]. SDS was added to the surfmers to give sufficient stability to the latexes. A mono-fluorooctyl maleate surfactant has been used to stabilize the polymerization of styrene in miniemulsion [57]. Although the polymerizable moiety was not fixed at the end of the fluorinated chain (the hydrophobe part), the authors claimed that the surfactant was copolymerized with the styrene monomer. They compared the IR spectra (vibration of –CF_2_ and –CF_3_) before and after dialysis and estimated that 92% of the surfactant remained grafted after dialysis.

Nanoparticles and nanocapsules from polymerization of styrene and/or divinylbenzene in miniemulsion could be produced in the presence of a polymerizable derivative of polyethylene oxide/polypropylene oxide which was used to stabilize the droplets [46]. XPS measurements on dialyzed samples confirmed the grafting of the polymerizable surfactant onto the surface of the particles. Matahwa et al. synthesized one cationic and one anionic polymerizable surfactant and copolymerized these with styrene and methyl methacrylate by RAFT in miniemulsion [58]. The rate of polymerization for the systems stabilized by the non-polymerizable surfactants was similar to systems for which polymerizable surfactants were employed. Cao et al. synthesized and measured the CMC of another cationic polymerizable surfactant and copolymerized it with styrene [59]. Fluorescent particles of polystyrene were created in miniemulsion by copolymerizing styrene, the cationic polymerizable surfactant *N,N*-dimethyl-*N*-*n*-dodecyl-*N*-2-methacryloyloxyethyl ammonium bromide, and eventually the polymerizable dye 1-pyrenylmethyl methacrylate [60]. The pyrene dye encapsulated in the particles displayed an excitation lifetime 17 times longer than pyrene dissolved in THF.

### Metal-catalyzed polymerizations

At the end of the last century, many groups focused their research on the production of polyolefins in aqueous media. Ethylene as one of the most industrially relevant monomers was polymerized via various heterophase polymerizations, including polymerization in miniemulsion. The first report on polymerization of ethylene in miniemulsion describes the synthesis of polyethylene nanoparticles in the presence of a nickel–ylide complex [10]. The catalyst was dissolved in toluene and hexadecane as hydrophobe and the solution was dispersed in an aqueous solution of surfactant (SDS). The mixture was homogenized and ethylene was subsequently added to the system. In this example, the homogenization was by simple mechanical stirring and the droplet size was not measured prior to polymerization. Therefore it is difficult to know if the experiment represented a “true” miniemulsion polymerization process. A similar process was employed but with homogenization of catalyst/hexadecane/toluene, which was performed with either ultrasonication or high-pressure homogenizer in order to obtain a stable miniemulsion [61]. High molecular weight polyethylene (140,000 g∙mol^−1^) could be obtained in stable latexes with particles having a hydrodynamic diameter between 90 and 330 nm. The same principle was used with various catalysts from commercial sources [62]. It was also possible to copolymerize ethylene and up to 3 mol % 1-butene. Small nanoparticles (~200 nm) could be obtained by ethylene polymerization with a nickel(II) keto–ylide complex with 10% solids content in direct miniemulsion [63]. The same group copolymerized ethylene and polar and non-polar α-olefins in miniemulsion with a *P*,*O*-chelated Ni(II) catalyst to obtain dispersions with up to 30% solid content [64]. The copolymerization of carbon monoxide with ethylene or 1-olefins with catalysts formed in situ from palladium(II) complexes gave aliphatic polyketones [65]. The catalyst activity was slightly higher as compared to non-aqueous polymerizations in methanol with the same catalysts.

The emulsion and miniemulsion processes were compared for the copolymerization of ethylene with vinyl acetate [66]. For batch processes, ethylene incorporation in the copolymer was found to be higher in miniemulsion than in emulsion due to the low solubility of ethylene in water and hence its poor transfer through the continuous phase. The use of semibatch processes reduced the difference between emulsion and miniemulsion polymerization in term of incorporation of ethylene in the copolymer. Instead of miniemulsified catalyst, functionalized polystyrene nanoparticles were synthesized in emulsion and miniemulsion by non-covalently immobilized metallocene catalysts [67]. The catalytic polymerization of butadiene with a cobalt catalyst was found to give highly crystalline 1,2-polybutadiene with a particle size of 150–200 nm [68]. The copolymerization of isoprene afforded low crystalline polymers. After Grubbs popularized water resistant catalysts for metathesis polymerizations, it became clear that metathesis could be also performed in aqueous heterophase systems. Claverie et al. studied the ring-opening metathesis polymerization (ROMP) in emulsion and miniemulsion [69]. Water-soluble ruthenium alkylidene was used for emulsion polymerization of norbornene, whilst an oil-soluble catalyst was employed for the miniemulsion polymerization of norbornene, 1,5-cyclooctadiene, cyclooctene. Similar to the polymerization of ethylene, which is described above, an organic solution of the catalyst was first miniemulsified in water and then the monomer was added to the miniemulsion. The monomer conversion was found to be moderate for the two latter monomers, and relatively high (97%) in the case of norbornene with an obtained particle size of 250 nm.

**Scheme 1:**
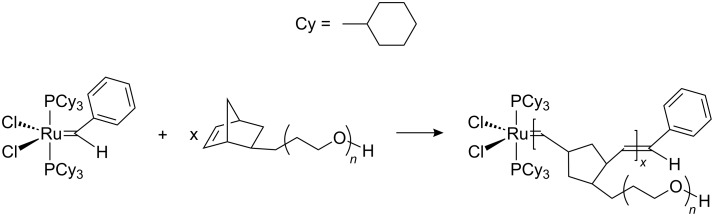
Synthesis of the macroinitiator for ROMP in direct miniemulsion [71].

Another group compared the dispersion, miniemulsion, and suspension polymerization for the ROMP of norbornene or cyclooctadiene [70]. For the miniemulsions, two approaches were followed, i.e., the addition of a catalyst solution to a miniemulsion of the monomer and the addition of monomer to miniemulsion of Grubbs catalyst in water. Although the first approach could yield simultaneously high conversion and stable latexes, particles with sizes above 400 nm without coagulum and 100% conversion could be obtained with the second approach. A water-soluble ruthenium carbene complex (PEO-based catalyst) was prepared as shown in Scheme 1 and used in the direct miniemulsion ROMP of norbornene [71]. Particles with sizes of 200–250 nm could be obtained. The catalytic polymerization of norbornene in direct miniemulsion was also carried out in the presence of an oil-soluble catalyst that was generated in situ, or with a water-soluble catalyst [72]. The reaction was faster when the oil-soluble catalyst was used.

Finally, helical substituted polyacetylene could be efficiently polymerized in direct miniemulsion to yield a latex displaying intense circular dichroism [73]. Particles from 60 to 400 nm could be prepared and the optical activity increased with decreasing particle size. Films were prepared from dried miniemulsion latexes which were then mixed with polyvinyl alcohol in order to preserve the optical activity.

### Ionic polymerizations

**Figure 4:**
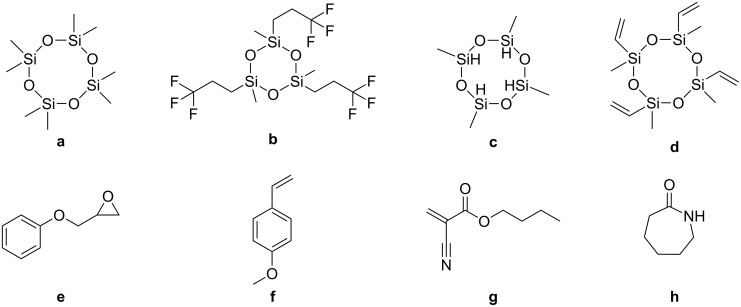
Monomers used in ionic miniemulsion polymerization. **a:** octamethylcyclotetrasiloxane [9,74], **b:** 1,3,5-tris(trifluoropropylmethyl)cyclotrisiloxane [75], **c:** 2,4,6,8-tetramethylcyclotetrasiloxane [76], **d:** tetramethyltetravinylcyclotetrasiloxane [77], **e:** phenyl glycidyl ether [78], **f:** *p*-methoxystyrene [79–81], **g:** *n*-butyl cyanoacrylate [82–90], **h:** ε-caprolactam [14].

Although different ionic polymerizations in heterophase have been reported in the literature, they are scarcely described and can be hence still considered as unconventional systems. Ionic miniemulsion polymerizations were carried out either under mild conditions (e.g., in the presence of water) or in water-free conditions (see Figure 4). Historically, ionic polymerizations in miniemulsion under mild conditions were investigated before miniemulsion polymerization requiring water-free conditions (published in 2005). The monomers polymerized for both types of polymerization are listed in Table 5.

**Table 5:** Monomers employed for ionic polymerizations in miniemulsion.

Monomer	Ref.

octamethylcyclotetrasiloxane	[9,74]
1,3,5-tris(trifluoropropylmethyl)cyclotrisiloxane	[75]
2,4,6,8-tetramethylcyclotetrasiloxane	[76]
tetramethyltetravinylcyclotetrasiloxane	[77]
phenyl glycidyl ether	[78]
*p*-methoxystyrene	[79–81]
*n*-butyl cyanoacrylate	[82–90]
ε-caprolactam	[14]

Cyclosiloxanes, for instance, can also be easily polymerized in direct miniemulsion. Octamethylcyclotetrasiloxane, [9,74], 1,3,5-tris(trifluoropropylmethyl)cyclotrisiloxane [75], 2,4,6,8-tetramethylcyclotetrasiloxane [76], and tetramethyltetravinylcyclotetrasiloxane [77] were polymerized in miniemulsion to yield polydimethylsiloxane, poly(trifluoropropylmethyl)siloxane, poly(methylhydrogenosiloxane), and multiblock vinyl functionalized silicones, respectively. Oligomers of phenyl glycidyl ether were produced in direct miniemulsion initiated by the counter anion of the surfmer didodecyldimethyl ammmonium hydroxide [78]. Cationic polymerization can also be performed in direct miniemulsion in the presence of water. *p*-Methoxystyrene was polymerized using the inisurf dodecylbenzenesulfonic acid with a monomer content up to 40 wt % [79]. The same group also investigated the same system in the presence of ytterbium triflate and found that inverse systems were formed [80]. The rate of polymerization was found to be slower than for the direct system whereas the molecular weights obtained were larger. The polymerization was initiated by 1-chloro-1-(*p*-methoxyphenyl)ethane (*p*-MOS-HCl) and catalyzed by trisdodecyl sulfate ytterbium, which is both a surfactant and a Lewis acid [81]. The Lewis acid surfactant did not play the expected role, since the *p*-MOS-HCl was hydrolyzed. The resulting hydronium ion protonated the SDS surfactant, which acted as an inisurf in the interfacial cationic polymerization process.

Alkyl cyanoacrylate monomers are probably the simplest monomers to polymerize anionically since the monomer is very reactive and the polymerization can be conducted in the presence of a large amount of water. Moreover, the polymers were shown to be able to pass the blood brain barrier, making them ideal candidates as vectors for drug delivery. Therefore it is not surprising that several groups have reported the polymerization of cyanoacrylates in miniemulsion. Limouzin et al. obtained low molecular weight oligo(*n*-butyl cyanoacrylate) (≤1,200 g∙mol^−1^) in the presence of a surfactant with a sulfonic acid group that slowed down the polymerization [82]. Altinbas et al. compared the polymerization of *n*-butyl cyanoacrylate both in macroemulsion and in miniemulsion in the presence of an oil (caprylic/capric triglyceride) with water as continuous phase [83]. The latter method yielded capsules with a higher stability. Solid-state NMR spectroscopy showed that the polymer was in contact with both water and oil, leading the authors to identify their particles as capsules (core–shell with oil as the core). Huang et al. showed that paclitaxel was encapsulated with high efficiency in poly(*n*-butyl cyanoacrylate) produced in miniemulsion [84]. Amino acids have been employed to initiate the polymerization of *n*-butyl cyanoacrylate in miniemulsion [85]. The nanoparticles were hence functionalized with carboxyl groups as shown by the pH dependence of the zeta potential. Functionalized nanoparticles of the same polymer were prepared by the polymerization of *n*-butyl cyanoacrylate in miniemulsion in the presence of methoxypoly(ethylene glycol) with the same oil used by Altinbas et al. [86]. Based on FT-IR and ^1^H NMR measurements, the authors claimed that poly(ethylene gycol) (PEG) chains were connected to the poly(*n*-butyl cyanoacrylate). The same approach has been used by other authors with other surface active initiators based on PEG [87]. The hydrophilic layer thickness and surface coverage of non-dialyzed nanoparticles were estimated for various surfactant concentrations [88]. The investigation was carried out by the same group with a modified dextran as stabilizer [89].

An unconventional approach was reported by Musyanovych and Landfester for the polymerization of *n*-butyl cyanoacrylate [90]. The monomer was solubilized in the continuous phase and the polymer precipitated during the polymerization at the interface of aqueous droplets. The process could be successfully used for the encapsulation of DNA with an encapsulation efficiency of almost 100%. The method is particularly interesting since it allows the encapsulation of hydrophilic substances in the polymer capsules.

The “classical” miniemulsions described above are, however, limited to particular monomers for anionic polymerization. In fact in many cases, the initiator and active species are sensitive to water and hence cannot be polymerized in water-in-oil or oil-in-water miniemulsions. In 2005 we reported the anionic polymerization of ε-caprolactam in non-aqueous miniemulsion polymerization [14]. Since the monomer ε-caprolactam is hydrophilic, the polymerization had to be carried out in inverse miniemulsions. ε-Caprolactam-in-oil miniemulsions could not be stabilized efficiently in contrast to DMSO-in-oil miniemulsions. Thus, ε-caprolactam was dissolved in DMSO to build the dispersed phase and polyamide-6 nanoparticles could be obtained. The synthesis strategy paved the way for various water-free reactions to be performed in the miniemulsion nanodroplets, including the formation of hydrophilic polyurethane capsules and particles as discussed below.

### Enzymatic polymerization

**Figure 5:**
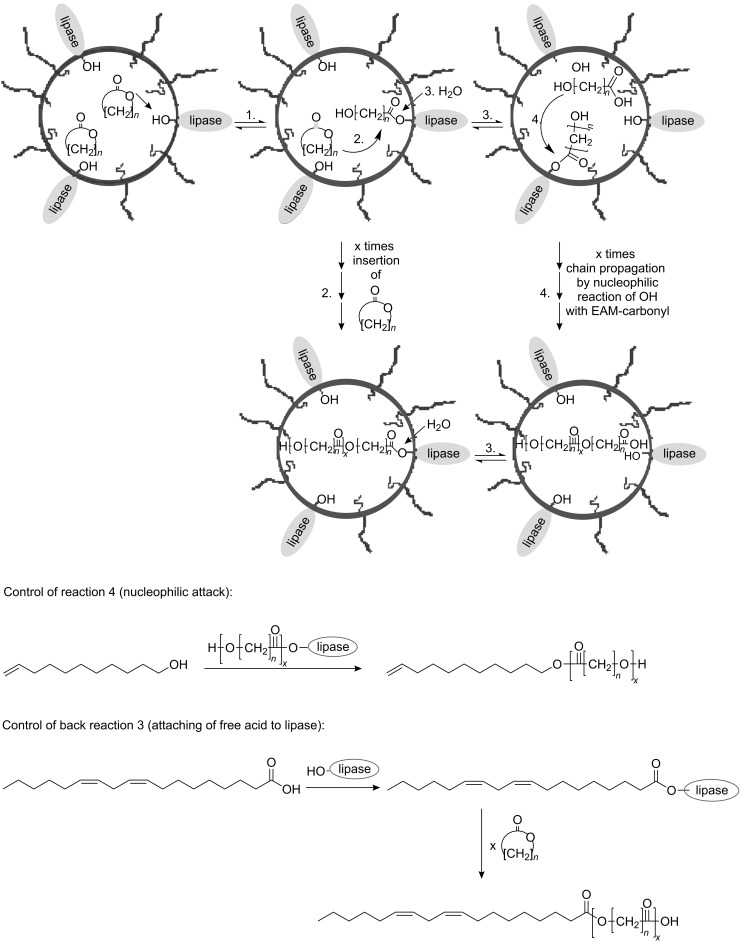
Enzymatic reactions in miniemulsion droplets (reproduced with permission from [91]. Copyright (2003) Wiley-VCH Verlag GmbH & Co, KGaA). EAM = enzyme-activated monomer.

Enzymatic polymerization could be successfully performed in direct miniemulsion with significant advantages compared to the traditional bulk process. In fact, the bulk process yields only low molecular weight polymers and conversions are limited to 80% after 5 days (see Figure 5). Polyesters were polymerized by enzymatic polymerization of pentadecanolide in direct miniemulsions with amphiphilic lipases (e.g., lipase-PS^TM^) [91]. Due to the very large interfacial area available, an apparent molecular weight as large as 200,000 g∙mol^−1^ could be obtained with full conversion of the monomer after 2 h.

Another advantage of miniemulsion systems compared to solution polymerization was demonstrated by Qi et al. who were able to polymerize styrene with horseradish peroxidase, hydrogen peroxide, and a β-diketone in aqueous direct miniemulsion [92]. Normally, only hydrophilic monomers can be polymerized with this initiating system in water or a co-solvent, e.g. THF, is required, however, the yields of polymer are low. Although the conversion to polymer was moderate in miniemulsion, polymers with apparent molecular weight of up to 406,000 g∙mol^−1^ could be obtained.

### Oxidative polymerization

**Figure 6:**
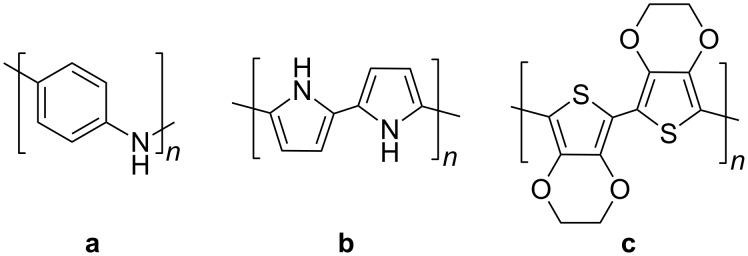
Chemical structure of **a:** polyaniline (leucoemeraldine), **b:** polypyrrole, **c:** poly(ethylene dioxythiophene).

Semiconducting polymers are usually difficult to process due to their low solubilities and therefore several groups have investigated polymerization in miniemulsion to improve their processibilities. Oxidative polymerizations were carried out in miniemulsion either in the droplets or on the surface of nanoparticles to create an additional shell. The monomers polymerized are shown in Figure 6.

Aniline and anilium hydrochloride were polymerized in direct and in inverse miniemulsion, respectively [93]. The polymerization of anilium hydrochloride was initiated by hydrogen peroxide and gave highly crystalline emeraldine polyaniline. In direct miniemulsions, additional stabilizers such as poly(vinyl pyrrolidone) or poly(vinyl alcohol) were employed to preserve colloidal stability. The polymerization of aniline in direct miniemulsion has also been reported by other authors [94]. After polymerization, they treated the polymer with stannous chloride and doped the polymer with *p*-toluenesulfonic acid. The conductivity was increased dramatically when stannous chloride was employed, which the authors attributed to the reduction of part of pernigraniline in the emeraldine base structure. Such oxidative polymerization of aniline can be used to add an additional conductive shell to preformed latexes. For instance, Li et al. polymerized aniline in the presence of dodecylbenzesulfonic acid on the surface of polyurethane and polyurethane/polymethyl methacrylate nanoparticles prepared in miniemulsion [95]. The same approach was previously reported for the polymerization of pyrrole initiated by iron(III) chloride on polystyrene latexes produced in miniemulsion to yield particles with sizes ranging from 50 to 70 nm [96]. The conductivity of pellets prepared from the particles was found to be 15.4 S∙cm^−1^. Core–shell morphologies could be identified by selectively dissolving the polystyrene core in THF. Ham et al. dispersed single-wall carbon nanotubes and pyrrole before oxidative polymerization of the monomer [97]. The electric properties of the composite were investigated for application as electrode material for a supercapacitor. Ethylene dioxythiophene (EDOT) has also been polymerized on polystyrene latex in miniemulsion [98].

### Polyaddition

Polyaddition and polycondensation reactions always yield functional polymers since the polymers produced are terminated with reactive functional groups. A higher degree of functionality is easily attained using monomers bearing additional reactive groups that do not participate in the step-growth polymerization. Polyaddition and polycondensation are probably the polyreactions for which miniemulsion systems are the most beneficial. An emulsion polymerization scenario where a mixture of micelles and monomer droplets coexist will probably yield polymer particles with a bimodal size distribution. The first polyadditions in miniemulsion carried out were the reactions of polyepoxides and hydrophobic diamines, bisphenols, and dimercaptans [11]. Stable latexes of epoxy resins could be obtained and apparent molecular weights up to 20,000 g∙mol^−1^ were measured. Another group reported the reaction between chitosan oligosaccharide with ethylene glycol diglycidyl ether to encapsulate paclitaxel in methylene dichloride-water miniemulsions [99]. The encapsulation was determined by HPLC to be between ~84% and ~92% depending on the ratio and amount of the monomers used, and could be re-dispersed in water after removal of the solvent for release studies. Ethylene glycol diglycidyl ether and L-lysine were polymerized via interfacial polyaddition in inverse miniemulsion [100]. The particles were found to be amphoteric and bear positive charges from secondary amine groups below pH = 8.7, whereas negative charges from the carboxylate groups of the amino acid units were noted above this pH. ssDNA could be trapped at lower pH and could be subsequently released by increasing the pH to 11.0.

The most common miniemulsion polyaddition synthesis is the formation of polyurethanes. Originally, there was considerable interest in producing water-borne polyurethane dispersions to replace the solvent-borne formulations and therefore much effort was expended in investigating the synthesis of polyurethane in aqueous miniemulsions. Hydrophobic diols such as 1,12-dodecanediol, bisphenol A, and/or neopentyl glycol, and the slow reacting isophorone diisocyanate (IPDI) were reacted in miniemulsion droplets [12]. Similar reactions were performed but in the presence of an organotin catalyst [101]. Relatively high apparent molecular weight polyurethane could be obtained. The molecular weight could be increased by the use of an organotin catalyst, a solvent in the dispersed phase and an excess of diisocyanate compared to diol. Instead of synthetic polyols, it is possible to employ polyols from renewable resources to synthesize polyurethane in miniemulsion. For example, castor oil (a triol) has been used as the monomer [102]. Li et al. showed that short diols can be also replaced efficiently by poly(tetramethylene glycol) [103]. The polyaddition reaction to form urethane bonds was also carried out with a cyclodextrin derivative and IPDI as the diisocyanate component [104]. The particles were used to encapsulate nimodipine, a calcium channel blocker.

A popular method to form polyurethane latexes in direct aqueous miniemulsions is to dissolve a reactive preformed prepolymer in the dispersed phase and subsequently carry out the polyaddition. The molecular weights obtained are usually higher for these two-step methods than for the one-pot method described above. Poly(propylene glycol) terminated polyurethane particles with IPDI were polymerized with a diol, an organotin catalyst, and a triol as crosslinker in miniemulsion [105]. Such prepolymers were also polymerized in the presence of monomers, which can be polymerized under radical conditions to yield hybrid latexes [106–107]. Li et al. showed that homogeneous or core–shell morphologies could be obtained depending on the diol added and on the location of the initiator (water-soluble versus oil-soluble) [107]. Another prepolymer used is polydimethylsiloxane terminated by hydroxy groups; this was reacted with IPDI to yield silicone/polyurethane hybrid latexes [108]. An interesting approach is to polymerize a monomer for polyaddition possessing an additional functionality. For instance, the acyl chloride of the azo-initiator 4,4'-azobis(4-cyanopentanoic acid) was reacted with 2,4-diethyl-1,5-pentanediol to yield a diol functionalized with an azo-bond [109]. The functionalized diol was subsequently polymerized with a diisocyanate to yield particles of cleavable polyurethanes. In a second step it was possible to cleave the azo-bonds and polymerize styrene in the nanodroplets. This approach hence combines free-radical polymerization and polyaddition for the production of hybrid block-copolymer particles. Polyurethanes can also be prepared in inverse miniemulsion if the monomers or prepolymers are sufficiently hydrophilic. Polymerizations in non-aqueous inverse miniemulsions are even possible as was previously demonstrated [79]. Polyurethanes free from any urea could be hence produced in non-aqueous inverse miniemulsions in a one-pot process [110]. In this case the miniemulsions of dimethylformamide in hexane were stabilized by a copolymer surfactant with isoprene and methyl methacrylate blocks. A shell of polymethyl methacrylate could be subsequently added after the polyaddition [111]. One of the techniques associated with step-growth polymerizations is the so-called interfacial polyaddition or polycondensation (Table 6). Since the surface generated by the miniemulsion droplets is extremely large, fast reactions are expected to occur in such systems. The formation of a thin film around the nanodroplets allow the creation of core–shell or capsular morphologies.

**Table 6:** Polymer obtained by interfacial polyaddition in miniemulsion.

Miniemulsion	Polymer obtained	References

direct	epoxy	[100]
	polyurea	[112]
	poly(urethane-urea)	[113–115]
	
inverse	epoxy	[116]
	polyurea	[116–118,120]
	poly(urethane-urea)	[116–119,122]
	polyurethane	[116]
	polythiourea	[116]
	crosslinked dextran	[116–118]
	crosslinked starch	[116,121]
	crosslinked polyethyleneimine	[116]

Polystyrene–polyurea core–shell particles were prepared by miniemulsifying the styrene and a hydrophobic diisocyanate monomer followed by the addition of a diamine to the miniemulsion, and then the radical polymerization of styrene [112]. The presence of the polyurea shell was shown to prevent migration of encapsulated dye. Torini et al. carried out the reaction between a diisocyanate dissolved in oil and a diol dissolved in water and added after miniemulsification of the first monomer in the aqueous continuous phase [113]. Owing to the side reaction with water, only an oligomer with molecular weight ranging from 500 to 3000 g∙mol^−1^ could be obtained. The same procedure was used by Johnsen et al. with a different polyol, i.e., propanetriol instead of 1,6-hexanediol [114]. A biocompatible hydrophobic liquid core, Myglyol 812 triglyceride, was used in the droplets to yield core–shell polyurethane/urea for the encapsulation of ibuprofen [115].

**Figure 7:**
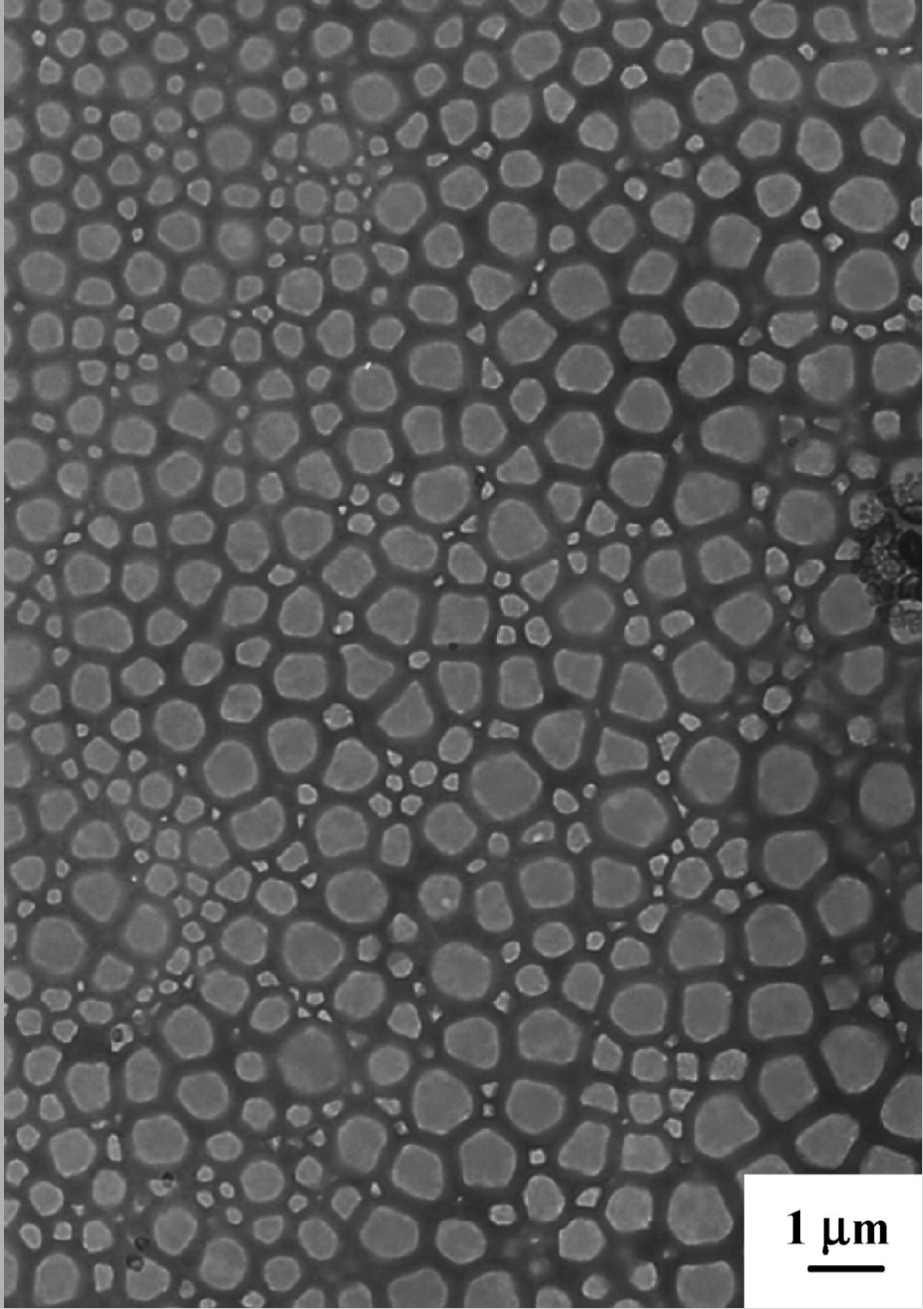
Transmission electron micrograph of polyurethane capsules synthesized by interfacial polyaddition in inverse miniemulsion (reprinted with permission from [116]. Copyright (2007) American Chemical Society).

Interfacial polyaddition in inverse miniemulsions is becoming especially popular since it allows the encapsulation of hydrophilic substances in various polymeric capsules. The method allows the formation of particles with capsular morphology consisting of a liquid core and a polymeric shell in comparison to the traditional monolithic morphology. Such capsular morphologies are suitable for drug delivery applications since the liquid in the core (e.g., water) has usually a higher solvent power than hydrophilic monomers. As for other techniques used to encapsulate drugs such as vesicle or liposome formation or solvent evaporation, the solvent used initially can be removed by dialysis or evaporation and exchanged with water to build the new dispersion medium. The capsules could be obtained with different wall thicknesses depending on the concentration of monomers and with a large variety of polymers, e.g., with epoxy, polyurethane (Figure 7), and polyurea, or based on synthetic polyamines, polythiourea, crosslinked starch, dextran or polyethylene imine [116]. Therefore, the functionality can be directly implemented in the capsules by using an excess of one of the monomers, or by using functionalized monomers or copolymers with additional functionality that do not participate in the polyaddition, in this case, e.g., amino, carboxylic, hydroxy, epoxide, isocyanate, or isothiocyanate.

Such capsules could be used to encapsulate contrast agents (Magnevist^®^, Gadovist^®^) for magnetic resonance imaging (MRI) in polyurethane, polyurea, and crosslinked dextran shells [117–118]. No significant difference in the relaxation time (*T*_1_) between the encapsulated agent and the contrast agent in solution could be detected therefore making the capsules good candidates for MRI. Fluorescent dyes as markers are suitable for particle–cell interactions and can be followed by LSM and FACS measurements. Thus, polyurethane/urea capsules produced in inverse miniemulsion were used to encapsulate a fluorescent dye with 90% efficiency [119]. Carboxymethylation was performed on the particle surface followed by physical adsorption of poly(2-aminoethyl methacrylate) or polyethylene imine polycations. As expected, the uptake of the capsules modified by the polycation was found to be higher than for non-modified capsules. Rosenbauer et al. used the same route but in the presence of a surfactant which crosslinked the shell [120]. The commercially available surfactant polyisobutylene-succinimide pentamine was reacted with the diisocyanate monomer. The capsule shell wall was found to be less permeable than for capsules synthesized with a non-crosslinkable surfactant. Baier et al. used the previously described synthesis to carry out a polymerase chain reaction (PCR) in crosslinked starch nanocapsules [121]. The permeability of the shell was also evaluated by fluorescence spectroscopy. The combination of cleavable polyurethane [109] with the interfacial polyaddition described above [116] afforded polymer shells that could be cleaved by UV-irradiation, temperature, or by pH change [122]. In order to study the release of encapsulated sulforhodamine dye from the capsules, polyurethane with and without cleavable functionalities were synthesized. Fluorescence spectroscopy of the supernatant obtained by the centrifugation of both polymer capsules submitted to different stimuli was recorded and the release of the dye was found to occur on different time-scales in the case of the cleavable shells, i.e., minutes for UV-irradiation, hours for a temperature increase, and days for a pH change.

### Polycondensation

**Figure 8:**
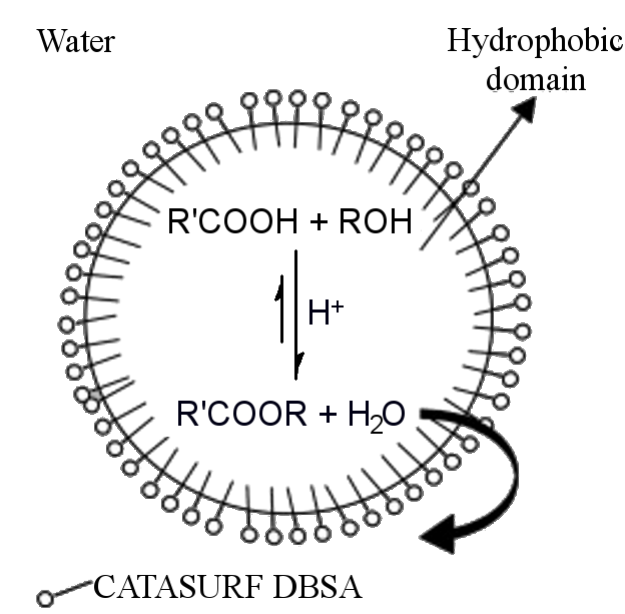
Schematics for the polycondensation reaction between hydrophobic alcohols and carboxylic acids surrounded by the aqueous continuous phase (not to scale for the surfactant) (reprinted with permission from [13]. Copyright (2003) Elsevier).

In aqueous miniemulsion, polycondensations are even more demanding than polyadditions since the water formed in the condensation reaction has to be transported away from the reaction locus. Barrere et al. showed that esterification polyreactions could be efficiently performed in aqueous miniemulsion droplets, even in the presence of a large amount of water (continuous phase), since the reaction locus (the droplets) are hydrophobic and become even more hydrophobic throughout the condensation reaction (Figure 8) [13]. The size of the droplets had no influence on the equilibrium, i.e., similar yields were obtained. Two major parameters were found to play a role in increasing the yield. The yield was higher if a) more hydrophobic monomers and b) diols with electron-donating groups were polymerized. The polycondensation of a diamine and sebacoyl chloride was carried out in direct miniemulsions in the presence of silica nanoparticles prepared in inverse microemulsion [123]. The polyamide was identified by infrared spectroscopy.

**Scheme 2:**
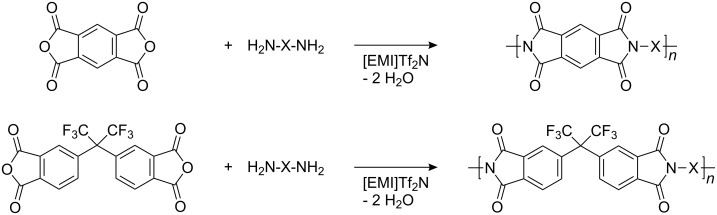
Polyimide from the reaction performed in the ionic liquid 1-ethyl-3-methylimidazolium bis(trifluoromethylsulfonyl)imide [EMI]Tf_2_N between a diamine H_2_N-X-NH_2_ and pyromellitic acid dianhydride, and 4,4'-(hexafluoroisopropylidene)diphthalic anhydride (from [15]).

Polyimide nanoparticles could be synthesized in ionic liquids at temperatures up to 190 °C even although water is produced during the polycondensation as shown in Scheme 2 [15]. Here again, the reaction locus is hydrophobic and spills out the water into the continuous phase. The ionic liquid 1-ethyl-3-methylimidazolium bis(trifluoromethylsulfonyl)imide was used both as continuous phase and as stabilizer due to its amphiphilic properties. The approach is interesting for synthesizing polymers, which require very high polymerization temperatures. Star copolymers of polyethylene glycol and polypropylene glycol were crosslinked in inverse miniemulsion via an esterification reaction with a dithiodicarboxylic acid to yield nanogels [124]. The disulfide bonds were subsequently cleaved by reduction to yield thiols, whereas the nanogels were stable in PBS solution.

### Radiation-induced polymerization

Miniemulsions initiated by ^60^Co γ-rays are reported in a separate paragraph because of their peculiarities. The γ-ray initiated miniemulsion polymerization was conducted to synthesize polystyrene particles [125]. The dose rate and the total absorbed total dose were found to affect the particle size of the latex particles. Y-like branched surfactant were synthesized and used for γ-ray miniemulsion polymerization at room temperature [126]. Polyurethane (2 wt %) was used as the hydrophobe in the miniemulsion polymerization of styrene and was sufficient to ensure a shelf-life of 1 year for the miniemulsion [127]. In both cases [126–127], the particle size and distribution were preserved throughout the polymerization. The copolymerization of styrene with 1-vinyl-2-pyrrolidone as polar monomer in the presence of dodecane in the oil droplets also gave nanocapsules [128]. ^1^H NMR spectroscopy measurements showed that graft copolymers were obtained by radiation-induced polymerization instead of random copolymers. Copolymers of butyl acrylate, acrylic acid, acrylonitrile, *N*-hydroxymethylacrylamide, and perfluoroalkylethyl methacrylate were prepared in direct miniemulsions with yields up to 96% within 34 h [129]. Functional polystyrene latexes were obtained by copolymerization of styrene with a polymerizable surfactant containing a carboxylic acid group [130]. The authors reported a narrowly particle size distribution when the monomers were polymerized by γ-rays compared to initiation with potassium peroxodisulfate. The presence of carboxylic acid groups at the surface of the particles was confirmed by X-ray photoelectron and FT-IR spectroscopy. Finally, graft hybrid copolymers of polyurethane and polymers from vinyl monomers have also been reported [131]. In a first step, the polyaddition reaction was carried out between a polybutadiene terminated with hydroxy groups and the IPDI monomer in aqueous direct miniemulsion. The grafting of vinyl monomers on the PU backbone was then induced by γ-ray irradiation.

### Coupling reactions

Pd-catalyzed cross-coupling reactions were carried out in direct aqueous miniemulsions with 1,2,4-tribromobenzene as crosslinker [132]. Aqueous latexes of crosslinked poly(*p*-phenylene ethynylene) were obtained and their opto-electronic properties were found to be similar to the linear polymer dissolved in toluene. The synthesis of fluorescent conjugated particles of poly(arylene diethynylenes) in direct miniemulsions by Glaser coupling has also been reported [133]. 4,4'-Dinonyl-2,2'-bipyridine was found to be a suitable ligand for solubilizing the copper(I) chloride catalyst in the toluene droplets. A solution of the monomers in toluene was mixed with the solution of the catalyst and then the reaction mixture was miniemulsified in an aqueous solution of a cationic surfactant. The miniemulsion was stirred for several days in the presence of air.

### Particles from coordination polymers

**Figure 9:**
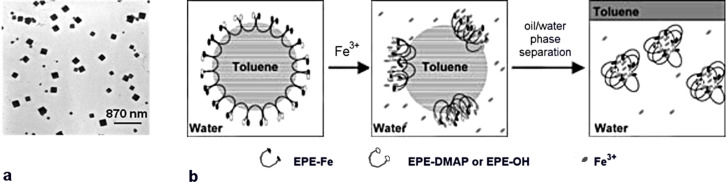
**a:** TEM micrograph of the cubic structures, **b:** proposed mechanism for the production of the nanocubes. The localized coordination polymerization is thought to destabilize the miniemulsion and nanocube growth is carried on in the aqueous phase (reproduced with permission from [136]. Copyright (2010) Wiley-VCH Verlag GmbH & Co, KGaA).

Prussian blue shells were created by adding iron(III) ions to direct miniemulsions of toluene/hexadecane stabilized by the organometallic surfactant [PEG-*b*-PPG-*b*-PEG-pentacyano(4-dimethylamino)pyridine) ferrate] [134]. Interparticle coordination was identified by electron microscopy and dynamic light scattering experiments. Nanoboxes could be formed when the concentration of surfactant was 4 wt % at a toluene content of 5 wt % [135]. Forty percent of the organometallic surfactant was replaced by PEG-*b*-PPG-*b*-PEG terminated by bromine atoms. When more 40% of the organometallic was replaced, the synthesis yielded only irregular structures due to inadequate crosslinking. PEG-*b*-PPG-*b*-PEG was used in combination with the organometallic surfactant to reduce the concentration of the latter surfactant on the droplet surface [136]. When 20 wt % toluene was used with low concentration of the organometallic surfactant, cubic nanoparticles were produced instead of the spherical shells (Figure 9). Only irregular structures were obtained in control experiments performed in water, i.e., without oil nanodroplets. The authors deduced that the initial confinement of the coordination polymerization hence played a significant role. The proposed mechanism for the formation of the cubic structures is shown in Figure 9.

## Conclusion

Compared to other heterophase polymerizations, miniemulsion polymerization offers an incomparable flexibility to create polymeric nano objects. Liquid or dissolved monomers can be polymerized by an unmatched variety of polymerization processes. The synthesis described above can be virtually extended to any polymerization or any polymer provided that the monomers can be emulsified, i.e., are not water- and oil-soluble. Even then, the monomers could be emulsified in sc-CO_2_ or in fluorinated solvents with a suitable surfactant.

## Acknowledgements

The image displayed as graphical abstract is a courtesy of the Empa, Switzerland, Laboratory for Protection and Physiology.
